# Recent advances in polymeric drug delivery systems

**DOI:** 10.1186/s40824-020-00190-7

**Published:** 2020-06-06

**Authors:** Yong Kiel Sung, Sung Wan Kim

**Affiliations:** 1 Department of Chemistry, College of Science, Dongguk University, Phildong-ro, Seoul, 04620 South Korea; 2 Department of Pharmaceutics and Pharmaceutical Chemistry, Center for Controlled Chemical Delivery, University of Utah, BPRB, Room 205, Salt Lake City, UT 84112 USA

**Keywords:** Drug delivery system, Polymeric drug delivery, Gene delivery system, Viral vectors, Non-viral vectors

## Abstract

**Background:**

Polymeric drug delivery systems have been achieved great development in the last two decades. Polymeric drug delivery has defined as a formulation or a device that enables the introduction of a therapeutic substance into the body. Biodegradable and bio-reducible polymers make the magic possible choice for lot of new drug delivery systems. The future prospects of the research for practical applications has required for the development in the field.

**Main body:**

Natural polymers such as arginine, chitosan, dextrin, polysaccharides, poly (glycolic acid), poly (lactic acid), and hyaluronic acid have been treated for polymeric drug delivery systems. Synthetic polymers such as poly (2-hydroxyethyl methacrylate), poly(N-isopropyl acrylamide)s, poly(ethylenimine)s, dendritic polymers, biodegradable and bio-absorbable polymers have been also discussed for polymeric drug delivery. Targeting polymeric drug delivery, biomimetic and bio-related polymeric systems, and drug-free macromolecular therapeutics have also treated for polymeric drug delivery. In polymeric gene delivery systems, virial vectors and non-virial vectors for gene delivery have briefly analyzed. The systems of non-virial vectors for gene delivery are polyethylenimine derivatives, polyethylenimine copolymers, and polyethylenimine conjugated bio-reducible polymers, and the systems of virial vectors are DNA conjugates and RNA conjugates for gene delivery.

**Conclusion:**

The development of polymeric drug delivery systems that have based on natural and synthetic polymers are rapidly emerging to pharmaceutical fields. The fruitful progresses have made in the application of biocompatible and bio-related copolymers and dendrimers to cancer treatment, including their use as delivery systems for potent anticancer drugs. Combining perspectives from the synthetic and biological fields will provide a new paradigm for the design of polymeric drug and gene delivery systems.

## Introduction

The research for polymeric drug delivery has been progressed for a long time since 1980’s [1–4]. The searches for new drug delivery systems approach and new modes of action represent one of the frontier research areas. Those involve multi-disciplinary scientific approaches to provide major advances in an improving therapeutic index and bioavailability at the specific delivery of drugs [5, 6]. Drug delivery system combines one or more traditional drug delivery systems with engineered technologies. The systems create the ability to specifically targeting point where a drug has released in the body and/or the rate at which it has released.

Biodegradable and bio-absorbable polymers make the magic possible choice for lot of new drug delivery systems. The bio-absorbable polymers like hydrogels such as poly (lactic acid) and poly (glycolic acid), and their copolymers have used to create the delivery component of the systems [7, 8]. Whether the drug delivery system relies on a biodegradable implant to deliver medicine subcutaneously or deep within the body, the biodegradable and bio-absorbable polymers provide a safe framework for delivering medicine without harm to the body.

Polymeric drug delivery system has defined as a formulation or a device that enables the introduction of a therapeutic substance into the body. It improves its safety and efficacy by controlling the rate, time, and place of release of drugs in the body. Drug delivery has achieved great development in the last two decades, but it remains a difficult task to regulate drug entry into the body such as brain. However, recent progress in studies of the carrier-mediated transportation of nano-drug delivery system across the blood-brain barrier is beginning to provide a rational basis for controlling drug distribution to the brain. The transport systems at the blood-brain barrier are the uptake transporters for natural nutrients such as amino acid, peptide, hexose, mono-carboxylate and stem cells [9–11].

The present paper has been reviewed for the polymeric drug and gene delivery systems of natural and synthetic polymers to formulate drugs into the backbone structures in various cases. The future prospects of the research for practical applications has been also proposed for the development in the fields.

### Natural polymers for drug delivery

#### Arginine derivatives

**Fig. 1:**
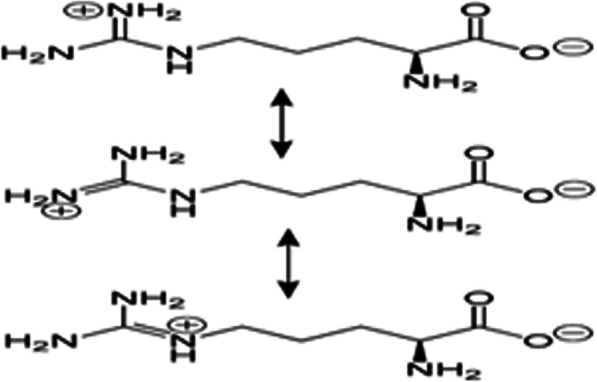
The delocalization of charge in guanidinium group of l-arginine for polymeric drug delivery systems

Arginine, also known as l-arginine, is α-amino acid that uses in the biosynthesis of proteins [12]. It contains α-amino group, α-carboxylic acid group, and a side chain consisting of a 3-carbon aliphatic straight chain ending in a guanidino group as shown in Fig. 1. At physiological pH, the carboxylic acid is deprotonated (−COO^−^), the amino group is protonated (−NH_3_^+^), and the guanidino group is protonated to give the guanidinium form (−C-(NH_2_)_2_^+^), making arginine a charged aliphatic amino acid [13]. The amino acid side-chain of arginine consists of a 3-carbon aliphatic straight chain, the distal end of which is capped by a guanidinium group, which has a pK_a_ of 12.48. It is therefore always protonated and positively charged at physiological pH. Because of the conjugation between the double bond and the nitrogen lone pairs, the positive charge is delocalized, enabling the formation of multiple hydrogen bonds in the chemical structures [14].

#### Chitosan derivatives

Chitosan is one of cationic polysaccharides derived from the natural chitin.

**Fig. 2:**
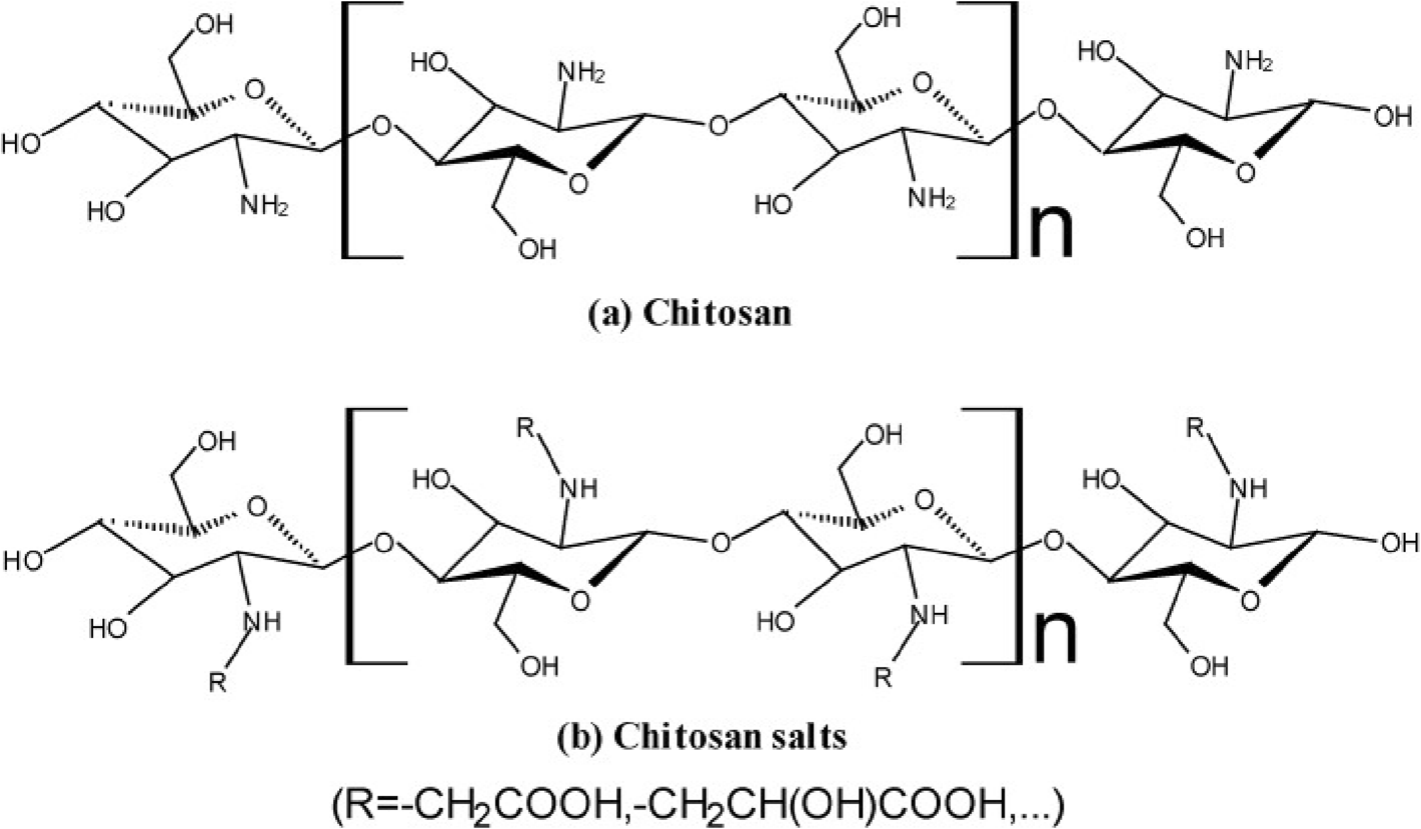
The chemical structures of chitosan (**a**) and chitosan salts (**b**)

As a cationic polymer with favorable property, it has been widely used to form polyelectrolyte complexes with polyanions for drug delivery [15, 16]. Chitosan is a linear copolymer composed by glucosamine and *N*-acteyl glucosamine units, via β-(1, 4) linkages, namely 2-amino-2-deoxy-β-d-glucan (Fig. 2a). Chitosan is the product of the deacetylation reaction of chitin (2-acetamido-2-deoxy-β-d-glucan). It has favorable biological properties such as nontoxicity, muco-adhesiveness, biocompatibility and the biodegradability [17–19]. The aqueous derivatives of chitosan such as chitosan salts (Fig. 2b), zwitterionic chitosan, and chitosan oligomers have drawn increasing attention due to their water-solubility for biomedical applications [20–23].

#### Cyclodextrin derivatives

**Fig. 3:**
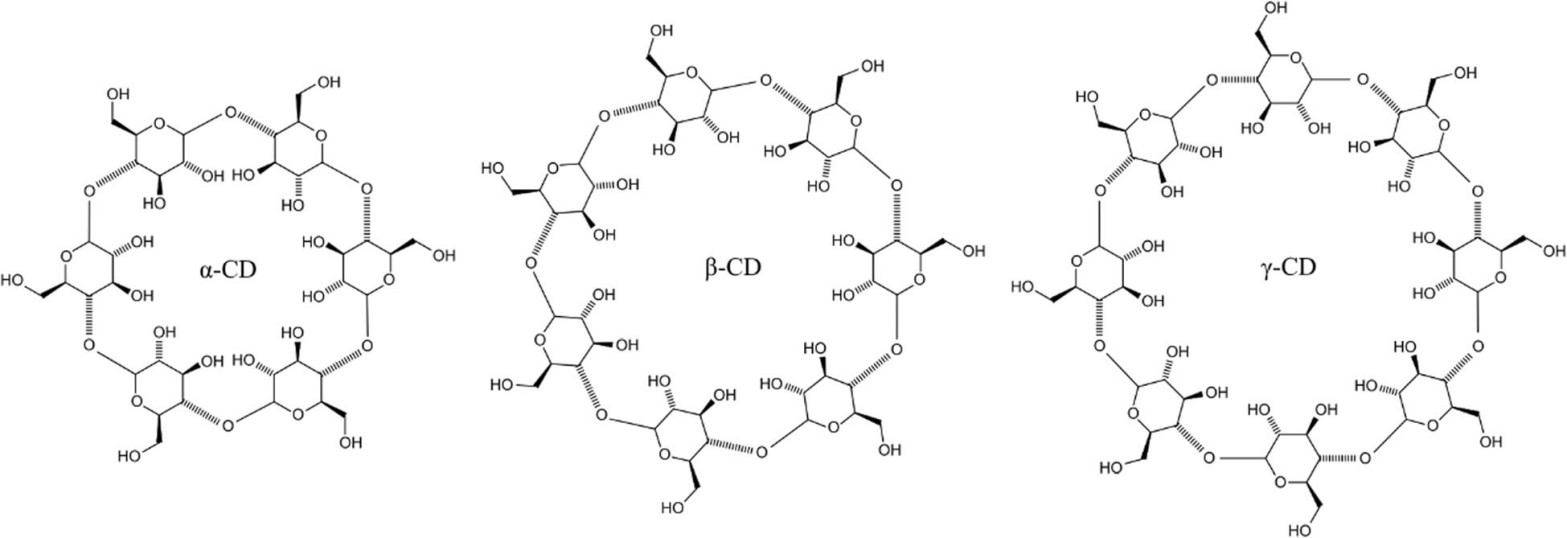
The chemical structure of the three main types of cyclodextrin (CD) for polymeric drug delivery systems

Cyclodextrin is a family of cyclic oligosaccharides composed of *α* (1, 4) linked glucopyranose subunits. Cyclodextrin is useful molecular chelating agent. There are three types of cyclodextrins in the nature. Those are named *α* (6 units), *β* (7 units) and *γ*-cyclodextrins (8 units) as shown in Fig. 3. *β*-Cyclodextrin is ideal for drug delivery due to the cavity size, efficiency drug complexation and loading, availability and relatively low cost [24]. An example of cyclodextrin in drug delivery system is 2-hydroxylpropyl derivate, which is a powerful solubilizer, and has a hydrophilic chain outside and a hydrophobic chain inside [25]. They are able to prevent the drug degradation and to improve the drug stability and solubility resulting on a higher bioavailability [26, 27]. Those are very useful for polymeric drug delivery systems for practical applications.

#### Poly (glycolic acid), poly (lactic acid), and hyaluronic acid

Glycolic acid is a useful intermediate for organic synthesis, in a range of reactions, including oxidation-reduction, esterification, and long chain polymerization. It has used as a monomer in the preparation of polyglycolic acid and other biocompatible copolymers. Two molecules of lactic acid have dehydrated to the lactone lactide. In the presence of catalysts, lactides polymerize to either atactic or syndiotactic polylactide which are biodegradable polyesters [28]. Glycolic acid and lactic acid have employed in pharmaceutical technology to produce water-soluble glycolate and lactate from otherwise-insoluble active ingredients. They have found further to use in drug delivery, topical preparations, and cosmetics to adjust acidity and for its disinfectant and keratolytic properties [29, 30]. Hyaluronic acid, which is a natural polymer, has the ability to target the CD44 over expressing cancer cells.

#### Polysaccharides

Natural polymers have been in use for many years with the aim of facilitating the efficiency of drugs and their delivery. Biodegradable polymers are widely being studied as a potential carrier material for specific drug delivery because of their non-toxic, biocompatible nature. Natural polysaccharides have investigated for application in drug delivery industry as well as in biomedical fields. Modified polymer has found its application as a support material for gene delivery, cell culture, and tissue engineering. Nowadays, natural polymers have modified to obtain novel biomaterials for controlled drug delivery applications.

**Fig. 4:**
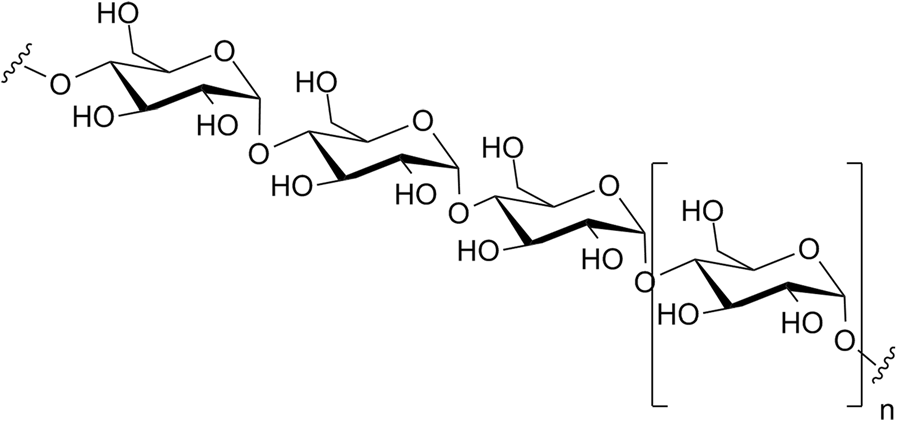
Amylose is a linear polymer of glucose mainly linked with α (1 → 4) bonds. It is one of the two components of starch polymer

Polysaccharides are long chains of carbohydrate molecules, specifically polymeric carbohydrates composed of monosaccharide units bound together by glycosidic linkages as shown in Fig. 4. This carbohydrate can react with water-hydrolysis using amylase enzymes at catalyst, which produces constituent sugars (monosaccharides or oligosaccharides). Natural saccharides are generally of simple carbohydrates called monosaccharides with general formula (CH_2_O)_*n*_ where *n* is three or more. Examples of monosaccharides are glucose, fructose, and glyceraldehyde [31]. Those natural polymers have used as biomaterials for drug delivery systems. Starch is a glucose polymer in which glucopyranose units have bonded by *alpha*-linkages. It has made up of a mixture of amylose and amylopectin. Amylose consists of a linear chain of several hundred glucose molecules and amylopectin is a branched molecule made of several thousand glucose units [32].

### Synthetic polymers for drug delivery systems

#### Poly(2-hydroxyethyl methacrylate)

Poly(2-hydroxyethyl methacrylate) [poly (HEMA)] is a polymer that forms a hydrogel in water or aqueous solution [33]. Poly (PHEMA) hydrogel for intraocular lens material was synthesized by solution polymerization using 2-hydroxyethyl methacrylate (HEMA) as raw material, azobisisobutyronitrile (AIBN), ammonium persulfate or sodium pyrosulfite (APS/SMBS) as catalyst, and ethyleneglycoldimethacrylate (EGDMA) or triethyleneglycoldimethacrylate (TEGDMA) as cross-linking additive [34]. Poly (HEMA) is commonly used to coat cell culture flasks in order to prevent cell adhesion and induce spheroid formation, particularly in cancer research. Older alternatives to pHEMA include agar and agarose gels [35, 36]. Equilibrium swelling, structural characterization and solute transports in swollen poly (HEMA) gels cross-linked with tripropyleneglycol diacrylate (TPGDA) were investigated for a wide range of TPGDA concentrations for drug delivery systems [37]. The physical and chemical properties of pilocarpine from poly (HEMA) hydrogels were investigated to elucidate the mechanism of drug–polymer interaction and the effect on drug release behavior of controlled release polymeric devices [38]. Poly (HEMA) hydrogels are widely used for biomedical implants. The extreme hydrophilicity of poly (HEMA) confers resistance to protein fouling, making it a strong candidate coating for ventricular catheters [39].

#### Poly(N-isopropyl acrylamide)s

Aqueous solution of poly(N-isopropyl acrylamide) (PNIPAAm) shows a lower critical solution temperature (LCST). The temperature-responsive polymer has investigated in the 1960’s [40]. They have established 32 C as the LCST of thermos-sensitive poly(N-isopropyl arylamide). The thermodynamic property of the system has evaluated from the phase diagram and the heat absorbed during phase separation by entropy effect [41]. The process of free radical polymerization for a single type of monomer, in this case of *N*-isopropyl-acrylamide, find to form the polymer known as a homo-polymerization. The initiator of azobisisobutyronitrile (AIBN) has commonly used in radical polymerization.

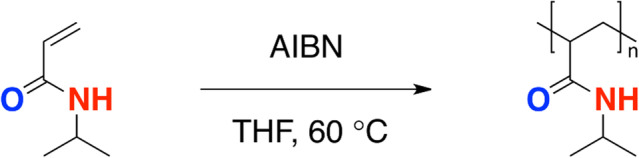



Thermo-responsive polymers have attracted much attention because of their potential biological and medical applications such as drug and gene delivery [42–44]. The swelling of cross-linked poly(*N*, *N*′-alkyl substituted acrylamides) in water was studied in relation to temperature changes. The thermo-sensitivity of water swelling has attributed to the delicate hydrophilic/hydrophobic balance of polymer chains and has affected by the size, configuration, and mobility of alkyl side-chain groups [45].

The cell culture surface of the polymer has readily prepared by the technique reversibly into hydrophilic and hydrophobic coatings of PNIPAAm-grafted polymers [46]. Temperature/pH sensitive hydrogels were prepared by copolymerizing N-isopropyl acrylamide (NIPAAm) and acrylic acid (AAc) [47]. The influence of polyelectrolyte on the LCST of temperature/pH sensitive hydrogels had investigated in the pH range of swelling ratio. The swelling ratio of the hydrogels in the presence of poly (allyl amine) (PAA) as a polyelectrolyte was also measured at the same conditions [48]. It has briefly discussed about the tumor micro-environmental responsive nano-particles in situ stimuli responsive such as pH, redox responsive, hypoxia sensitive, etc.

#### Poly (ethylenimine)s

**Fig. 5:**
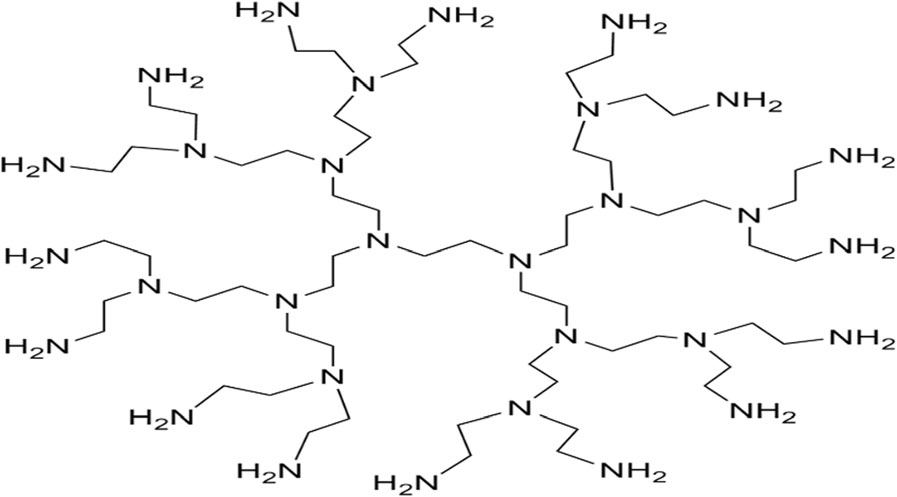
The chemical structure of poly (ethylenimine) s for polymeric drug delivery

Linear poly (ethylenimine)(PEI) is soluble in hot water, at low pH, ethanol or chloroform. They are insoluble in cold water, acetone, benzene, and ethyl ether. Branched PEI has synthesized by the ring opening polymerization of aziridine as shown in Fig. 5. Linear PEI is available by post-modification of other polymers like poly (2-oxazolines) or *N*-substituted polyaziridines [49]. Linear PEI was synthesized by the hydrolysis of poly (2-ethyl-2-oxazoline) [50, 51].

#### Poly(N-(2-hydroxypropyl) methacrylamide)s

Degradable diblock and multiblock (tetrablock and hexablock) N-(2-hydroxypropyl) methacrylamide (HPMA) copolymer-gemcitabine (GEM) and -paclitaxel (PTX) conjugates had synthesized by reversible addition-fragmentation chain-transter (RAFT) copolymerization followed by click reaction for preclinical investigation [52]. Poly (HPMA) copolymer-cytarabine and GDC-0980 conjugates were synthesized. In vitro studies demonstrated that both conjugates had potent cytotoxicity and their combination showed strong synergy, suggesting a potential chemotherapeutic strategy [53]. Telechelic water-soluble HPMA copolymers and HPMA copolymer-doxorubicin (DOX) conjugates had synthesized by RAFT polymerization mediated by a new bi-functional chain transfer agent that contained an enzymatically degradable oligopeptide sequence [54, 55].

#### Dendritic polymers

Dendritic polymers are highly branched polymers with controllable structures, which possess a large population of terminal functional groups, low solution or melt viscosity, and good solubility. Their size, degree of branching and functionality can be controlled and adjusted through the synthetic procedures. The research of dendrimer has increased on the design and synthesis of biocompatible dendrimer and its application to many areas of bioscience including drug delivery, immunology and the development of vaccines, antimicrobials and antivirals [56, 57].

**Fig. 6:**
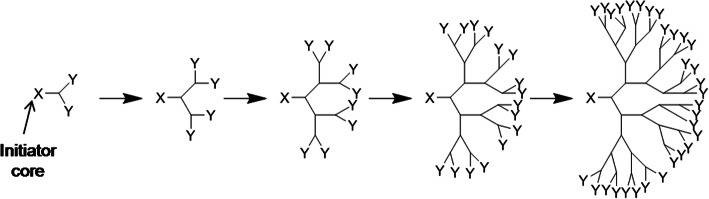
The schematic design of divergent synthesis of dendrimers for drug delivery

**Fig. 7:**
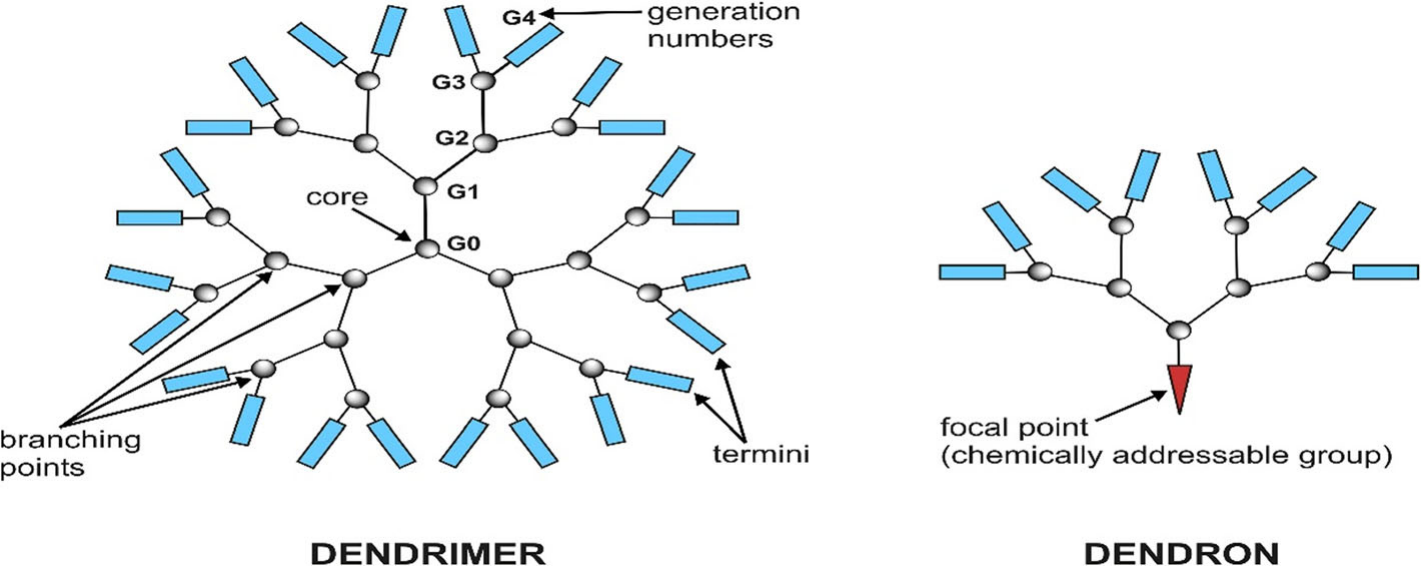
The chemical structures of dendrimer and dendron for drug delivery

The dendrimers are the members of a versatile, new class of polymer architectures, dendritic polymers after traditional linear, cross-linked, and branched types as shown in Fig. 6 and Fig. 7. The dendrimer type of bio-reducible polymer for efficient gene delivery had been also investigated [58].

#### Biodegradable and bio-absorbable polymers

**Fig. 8:**
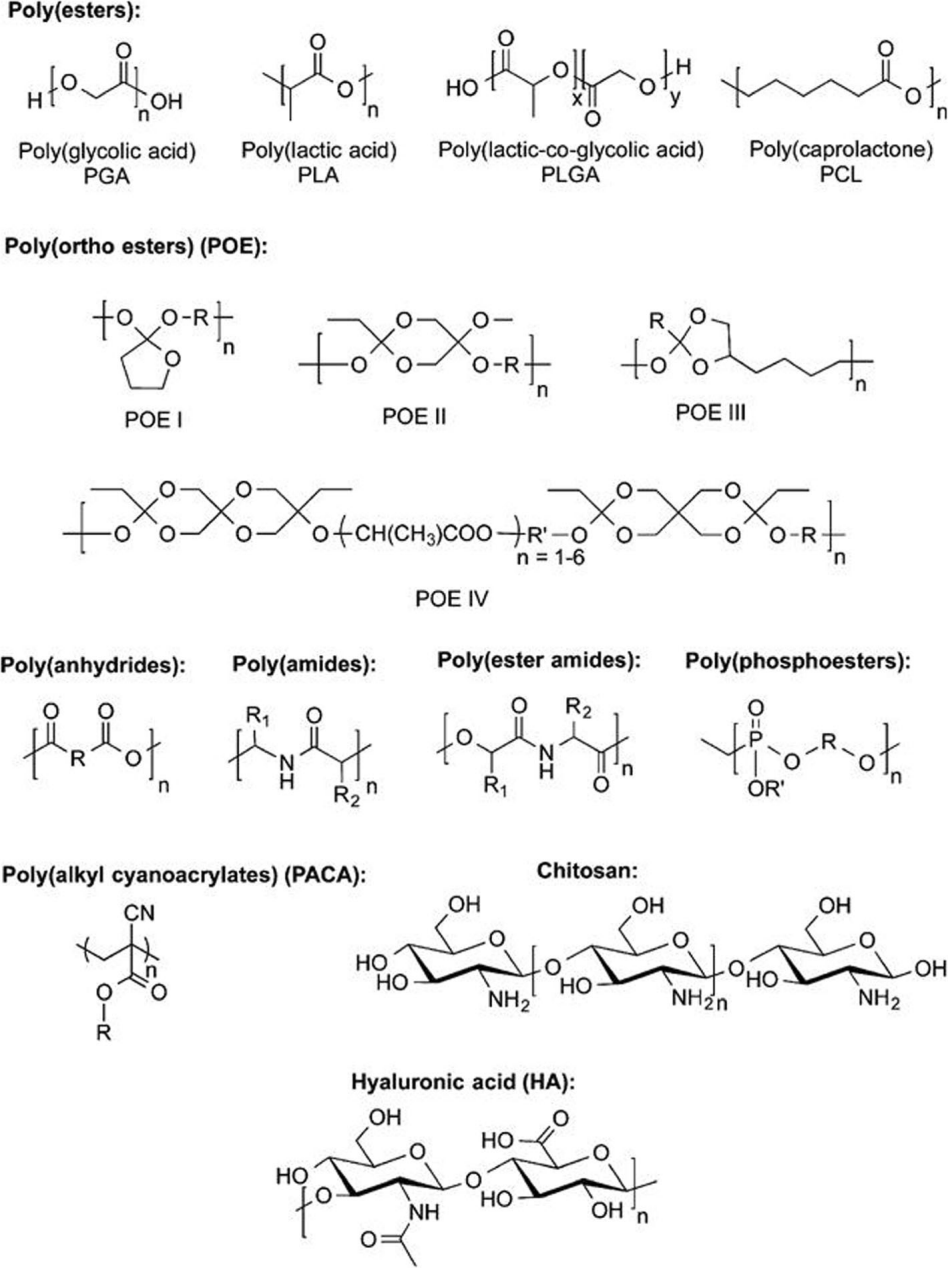
Biodegradable polymers with representative monomer units for polymeric drug delivery

Bio-absorbable drug delivery systems are a better choice for the application of drug carriers where only the temporary presence of the implant is needed [59]. Among the synthetic and biodegradable polymers, aliphatic polyesters such as poly (glycolic acid), poly (lactic acid), poly (caprolactone) and polydioxanone, are most commonly used and applied to drug delivery systems. As shown in Fig. 8, the several classes of polymers such as poly (esters), poly (ortho esters), polyanhydrides, and biodegradable polycarbonates have also been introduced as potential implant materials for drug delivery [60–62].

Biodegradable polymers commonly used include the *α*-hydroxy acids, polyanhydrides, poly (amides), poly (ester amides), poly (phosphoesters), poly (alkyl cyanoacrylates), poly (hyarulonic acids) and natural sugars such as chitosan, in addition to many other types of degradable polymers as shown in Fig. 7 Synthetic biodegradable polymers are favored in drug delivery systems, as they have immunogenicity as compared to biodegradable polymers from natural polymers [63–65].

### Polymeric drug delivery systems

#### Targeting polymeric drug delivery

The therapeutic targeting of biomimetic chitosan-PEG-folate-complexed oncolytic adenovirus has examined for active and systematic cancer gene therapy [66]. The oncolytic adenovirus coated with multi-degradable bio-reducible core-cross-linked poly (ethyleneimine) for cancer gene therapy had been also applied [67]. Hepatoma targeting peptide conjugated bio-reducible polymer complexed with oncolytic adenovirus for cancer gene therapy were investigated [68]. Despite considerable advances in tumor-targeting technologies, the lack of selectivity towards tumor cells is still the primary limitation of current cancer therapies. A novel strategy for targeted drug delivery to cancer cells had developed through the formation of a physical conjugate between doxorubicin (Dox) and the A10 RNA aptamer that binds to the prostate-specific membrane antigen (PSMA) [69].

The effective polymers have designed specifically for gene delivery, and much has learned about their structure–function relationships. With the growing understanding of polymer gene-delivery mechanisms and continued efforts of creative polymer scientists, it is likely that polymer-based gene-delivery systems will become an important tool for human gene therapy [70].

Nanoparticle-based therapeutics in lung cancer is an emerging area and covers the diagnosis, screening, imaging, and treatment of primary and metastatic lung tumors. Innovative engineering on polymeric nano-carriers allows multiple anticancer drugs and gene delivery to site-specific targets [71]. The targeted drug delivery and gene therapy through natural biodegradable nanoparticles is an area of major interest in the field of nanotechnology and pharmaceuticals [72].

#### Biomimetic and bio-inspired polymers

The biomimetic and bioinspired systems improve biocompatibility during drug delivery application. The success of such a drug delivery system depends on parameters like shape, surface, texture, movement, and preparation methods. The systems have great influence on the biological systems owing to their less toxicity, high biocompatibility, significant interaction, and so on [73–75]. The novel developments of dendritic polymers based targeting nanoscale drug delivery vehicles described here provide great potential to achieve better therapeutic indexes in cancer therapy as well as low side effect [76–78]. Although synthetic drug carriers have developed for many applications, it remains important to examine natural particulates, which range from pathogens to mammalian cell’s mechanisms. Biocompatible polymeric nanoparticles are considerably promising carrier candidates in delivery of drugs and genes because of their unique chemical and physical properties [79, 80].

#### Drug-free macromolecular therapeutics

Drug-free macromolecular therapeutics induce apoptosis of malignant cells by the crosslinking of surface non-internalizing receptors. The receptor crosslinking has mediated by the bio-recognition of high-fidelity natural binding motifs. Those have grafted to the side chains of polymers or attached to targeting moieties against cell receptors. This approach features the absence of low-molecular-weight cytotoxic compounds. Macromolecular therapeutics, also referred to as polymeric nano-medicines, are a diverse group of drugs characterized by their large molecular weight (MW), including polymer-drug conjugates, polymeric micelles, and polymer-modified liposomes [81–83].

### Polymeric gene delivery systems

Gene therapy is a promising new technique for treating cancer and.

genetic disorders by introducing foreign genomic materials into host cells to elicit a therapeutic benefit. The gene therapy has a potential in treating many diseases such as infectious disease and immune system disorders. The efficient delivery of therapeutic gene to target a cell is the most important step in gene therapy [84, 85]. Successful gene therapy is thus dependent on the development of an efficient delivery vector. There are non-viral vectors and viral vectors for gene delivery [86]. Pulmonary drug and gene delivery to the lung represents a non-invasive avenue for local and systemic therapies. Nano-sized particles offer novel concepts for the development of optimized therapeutic tools in pulmonary research. Polymeric nano-carriers are generally preferred as controlled pulmonary delivery systems due to prolonged retention in the lung [87].

#### Non-viral vectors for gene delivery

##### Polyethylenimine derivatives

Polyethylenimine (PEI) is a class of cationic polymers proven to effect for gene delivery [88]. Branched poly (ethylenimine)(PEI) 25 kDa is an efficient gene delivery vector with outstanding gene condensation ability and great endosome escape activity [89]. A bio-reducible polyethylene-imine (PEI (−s-s-)) was derived from low molecular weight PEI (1.8 kDa) for efficient gene delivery. The bio-reducible core molecules have expected to increase molecular weights and reduce the cytotoxicity of the copolymers. PEI (−s-s-) polyplexes showed higher transfection efficiency and lower cytotoxicity compared to branched PEI 25 kDa, Lipofectamine® 2000. In addition, PEI (−s-s-) derivatives (16 kDa) had formed stable polyplexes with a zeta-potential value of + 34 mV and the size of polyplex 61 nm [90]. The cytotoxicity of polyethylenimine (PEI) is a dominating obstacle to its application. Polyethylenimine (PEI) is a well-known cationic polymer, which has high transfection efficiency owing to its buffering capacity. It has reported that PEI is cytotoxic in many cell lines and non-degradable. In order to solve the problems, the polyethylenimine copolymers have introduced firstly in gene delivery systems [91].

#### Polyethylenimine copolymers

The introduction of poly (ethylene glycol) (PEG) blocks to PEI is one of the.

strategies to alleviate the cytotoxocity of PEI. However, it has well known that the transfection efficiency of PEGylated PEI has decreased to some extent compared to the corresponding PEI. Novel ABA triblock copolymers consisting of low molecular weight linear polyethylenimine (PEI) as the A block and poly (ethylene glycol) (PEG) as the B block were prepared and evaluated as polymeric transfectant. The PEI-PEG-PEI triblock copolymers displayed also an improved safety profile in comparison with high molecular weight PEIs. The linear PEI-PEG-PEI triblock copolymers are an attractive novel class of non-viral gene delivery systems [92].

Polyethylenimine-*alt*-poly (ethylene glycol) copolymers had been synthesized for an ideal gene carrier both safety and transfection efficiency. The copolymers were complexed with plasmid DNA. The resulting complexes exhibited no cytotoxic effects on cells even at high copolymer concentration. It’s transfection efficiency was influenced by poly (ethylene glycol)(PEG) molecular weight. The transfection efficiency was higher than that for PEI 25 K in HepG2 and MG63, whereas it was lower than that for PEI 25 K in HeLa cells [93].

Aiming to prepare a biodegradable gene vector with high transfection efficiency and low cytotoxicity, it had conjugated low molecular weight (LMW) PEIs to the biodegradable backbone polyglutamic acids derivative (PEG-b-PBLG) by aminolysis to form PEIs combined PEG-*b*-PLG-*g*-PEIs [94]. A series of tri-block co-polymers, PEG-*g*-PEI-*g*-poly (dimethylaminoethyl L-glutamine) (PEG-*g*-PEI-*g*-PDMAEG), as novel vectors for gene therapy was synthesized and evaluated [95]. The synthesized PEG-g-PEI-g-PDMAEG tri-block copolymers are promising candidates as non-viral carriers for gene delivery.

##### Polyethylenimine conjugated bio-reducible polymers

In order to introduce the disulfide bond between poly (cystamine-*bis*- (acrylamide) diaminohexane) [poly (CBA-DAH)] and PEI 1.8 kDa, Traut’s reagent were used to synthesize the products [96]. Poly (CBA-DAH)-PEI can be confirmed its potential as a gene delivery carrier. For the identification of the products, the proton peaks of poly (CBA-DAH) and PEI were shifted downfield due to steric hindrance caused by the conjugation between P (CBA-DAH) and PEI. In addition, the conjugation ratio of PEI to the PCDP has been calculated by the ratio of the integration of the proton spectra peaks in poly (CBA-DAH)(−NCH_2_CH_2_CH_2_CH_2_-CH_2_CH_2_NH_2_) and CH_2_ of PEI. Poly (ethylenimine) (PEI, 1.8 kDa) was conjugated to poly (CBA-DAH) via a disulfide bond. The PEI conjugated poly (CBA-DAH)[PCDP] was able to bind with pDNA at a very low molecular weight ratio and form the polyplexes with nano-size and positive surface charge.

The PCDP polyplexes show 10 times higher gene transfection efficiency than Lipofectamine® polyplexes in bio-mimic in vivo condition. The bio-reducible PEI (1.8 kDa) conjugated poly (CBA-DAH) is finally concluded as an efficient polymeric gene delivery carrier [97, 98]. It has been concluded that the PEI(1.8 kDa)-PCDP synthesized in our laboratory is one of the good candidates as mRNA, siRNA, and pDNA carriers for efficient gene delivery systems [99]. Outstanding representatives of bio-polymers that have emerged over the last decade to be used in gene therapy are synthetic bio-reducible polymers such as poly(l-lysine), poly(l-ornithine), linear and branched polyethyleneimine, diethyl-aminoethyl-dextran, poly (amidoamine) dendrimers, and poly (dimethyl-aminoethyl methacrylate) [100].

#### Viral vectors for polymeric gene delivery

Viral vectors not only have the ability to effectively infect cells, but also transfer DNA to the host without causing an immune response. Viral vectors have designed to be safe by making them incapable of replication. Gene transferred by viral vector has dominated the clinical trials in gene therapy, because they are more efficient than physicochemical methods [101]. Viral vectors have divided into two types, which are integrating and non-integrating viral vectors. Integrated viral vectors have integrated into the human genome, including adeno-associated virus and retroviral vectors; non-integrating vectors, like adenoviral vectors. They remain in the nucleus without having integrated into the chromosomal DNA and RNA. Gene delivery systems for gene therapy provide a great opportunity for treating diseases from genetic disorders, cancer, and other infections. The recent development of gene delivery system has reviewed for viral delivery systems and non-viral delivery systems [102].

##### DNA conjugates

Gene therapy is a promising new technique for treating many serious incurable diseases such as cancer and genetic disorders. The main problem limiting the application of this strategy in vivo is the difficulty of transporting large, fragile and negatively charged molecules like DNA into the nucleus of the cell without degradation [103]. The gene therapy of DNA conjugate is as a new promising technique used to treat many incurable diseases and the different strategies used to transfer DNA, taking into account that introducing DNA into the cell nucleus without degradation. It is essential for the success of this therapeutic technique.

The use of DNA as a drug is both appealing and simple in concept. In many instances, the feasibility of such an approach has been established using model systems. In practical terms, the delivery of DNA to human tissues presents a wide variety of problems that differ with each potential therapeutic application [104]. The challenge for the therapeutic use of viral vectors is to achieve efficient and often extended expression of the exogenous gene while evading the host defenses. Recent engineering of modified viral vectors has contributed to improved gene delivery efficacy [105]. The design of polymeric nanoparticles for gene therapy requires engineering of polymer structure to overcome multiple barriers, including prolonged colloidal stability during formulation and application. Poly(β-amino ester) s have been shown effective as polymeric vectors for intracellular DNA delivery [106].

##### RNA conjugates

Most of the current methods for programmable RNA drug therapies are unsuitable for the clinic due to low uptake efficiency and high cytotoxicity. RNA therapeutics including small-interfering RNAs (siRNAs), antisense oligonucleotides (ASOs), and CRISPR–Cas9 genome editing guide RNAs (gRNAs) are emerging modalities for programmable therapies that target the diseased human genome with high specificity and great flexibility [107]. RNA interference (RNAi) mediated gene silencing holds significant promises in gene therapy. A major obstacle to efficient RNAi is the systemic delivery of the therapeutic RNAs into the cyto-plasma without having trapped in intracellular endo-lysosomes [108].

RNA interference (RNAi) has been proven to be an useful approach to treat various genetic diseases. It can down-regulate specific protein expression by silencing the activity of its targeted gene [109, 110]. RDG could tightly condense shRNAs into stable complex nanoparticles. The RDG/shRNA nanoparticle had found to be highly selective in targeting the U-87 MG-GFP cells with over-expressed αvβ3 integrins via receptor-mediated endocytosis. The RDG/shRNA complex, which combines RGD-mediated active targeting and glutathione-triggered intracellular release and low cytotoxicity, appears to be a highly promising non-viral vector for efficient RNA delivery and therapy [111, 112]. Exosomes, unlike other vectors for gene delivery, present unique advantages such that exosomes are a cell-free natural system for ferrying RNA between cells, robust exosomal membrane can protect the RNA/gene of interest from digestion, and exosomes are rapidly taken up by target cells making them a more efficient vehicle for gene delivery [113]. Delivery of these miRNA molecule enriched-exosomes subsequently results in highly efficient overexpression or deletion of the designated miRNAs in the recipient cells both in vivo and in vitro [114].

## Conclusion and future prospects

The development of drug delivery carriers based on natural and synthetic polymers are rapidly emerging field. It takes advantages of the remarkable delivery mechanism, which has used by pathogens and mammalian cells, such as selective targeting and prolonged circulation by evasion of the immune systems. The biomimetic and bio-inspired systems have a bright future ahead with a lot of potentials to solve any obstacles encountered in polymeric drug delivery. The fruitful progress will have made in the application of biocompatible and bio-related copolymers and dendrimers to cancer treatment, including their use as delivery systems for potent anti-cancer drugs such as *cis*-platin and doxorubicin. The unique properties of dendrimers such as their high degree of branching, multi-valence, globular architecture, and well-defined molecular weight make them promising new scaffolds for polymeric drug delivery systems.

The micro-processes that are required for the development of such carriers, such as genetic engineering or in vivo treatments to incorporate therapeutic substances, make it difficult to maintain the integrity of natural and synthetic polymers with cells in a body. The gap between synthetic and biological systems has traditionally been very large. Recent advances in the synthesis of novel biomaterials and understanding of biological systems have paved the way towards bridging this gap. Polymeric drug delivery carriers that have based on pathogens such as bacteria and viruses are potentially immunogenicity for human body. A certain degree of immunogenicity can be ideal if pathogen-based carriers have intended for vaccine delivery, owing to their adjuvant ability. Combining perspectives from the synthetic and biological fields will provide a new paradigm for the design of polymeric drug delivery systems in near future.

## Data Availability

Not applicable.

## Abbreviations

AAc: Acrylic acid
AIBN: Azobisisobuyronitrile
APS/SMBS: Ammonium persulfate or sodium pyrosulfite
CD: Cyclodextrin
EGDMA: Ethyleneglycol dimethacrylate
DOX: Doxorubicin
GEM: Gemcitabine
HEMA: Hydroxyethyl methacrylate
HPMA: N-(2-hydroxypropyl) methacrylamide
LCST: Lower critical solution temperature
LMW: Low molecular weight
MW: Molecular weight
NIPAAm: N-isopropyl acrylamide
PAA: Poly (allyl amine)
PCDP: Poly (CBA-DAH)
PDMAEG: Poly (dimethylaminoethyl L-glutamine)
PEG-*b*-PLG-*g*-PEIs: Poly (ethylene glycol-*block*- poly(L-glutamate)-graft-poly (ethylenimine)s
PEG-*g*-PEI-*g*-PDMAEG: Poly (ethylene glycol-*graft*-poly (ethylenimine)- *graft*-poly (dimethyl amino ethyl L-glutamate)
PEI: Poly (ethylenimine)
PEG: Poly (ethylene glycol)
PNIPAAm: Poly(N-isopropyl acrylamide)
poly (CBA-DAH): Poly (cystamine-*bis*-(acrylamide) diaminohexane
poly (HEMA): Poly (2- hydroxyethyl methacrylate)
poly (HPMA): Poly(N-(2- hydroxypropyl) methacrylamide)
PSMA: prostate-Specific membrane antigen
PTX: Paclitaxel
TEGDMA: Triethyleneglycol dimethacrylate
TPGDA: Tripropylene glycol diacrylate

## References

[1] Anderson JM, Kim SW (1989). Advances in Drug Delivery Systems (3), Book Review. J Pharm Sci.

[2] Langer R, Peppas NA (2003). Advances in biomaterials, drug delivery, and bionanotechnology. AICHE J.

[3] Heller A (2005). Integrated medical feedback systems for drug delivery. AICHE J.

[4] Martinho N, Damgé C, Pinto C (2011). Reis, Recent advances in drug delivery systems. J Biomater Nanobiotechn.

[5] Din F, Aman W, Ullah I, Quereshi OS, Mustapha O, Shafique S, Zeb A (2017). Effective use of nano-carriers as drug delivery systems for the treatment of selected tumors. Int J Nanomedicine.

[6] Tiwari G, Tiwari R, Sriwastawa B, Bhati L, Pandey S, Pandey P, Bannerjee SK (2012). Drug delivery systems: An updated review. Int J Pharm Investig.

[7] SinhaVR LK (1998). Bio-absorbable polymers for implantable therapeutic systems, Drug Dev. Ind Pharm.

[8] Basua A, Kundurua KR, Doppalapudib S, Domba AJ, Khanb W (2016). Poly (lactic acid) based hydrogels. Adv Drug Deliv Rev.

[9] Teleanu DM, Chircov C, Grumezescu AM, Volceanov A, Teleanu RI (2018). Blood-brain delivery methods using nanotechnology. Pharmaceutics.

[10] Cacciatore I, Ciulla M, Fornasari E, Marinelli L, Di Stefano A. Solid lipid nanoparticles as a drug delivery system for the treatment of neurodegenerative diseases, Expert. Opin Drug Deliv. 2016;13(8):1121–31. 10.1080/17425247.2016. [Google Scholar]. 10.1080/17425247.2016.1178237 27073977

[11] Patel M, Souto EB, Singh KK (2013). Advances in brain drug targeting and delivery: limitations and challenges of solid lipid nanoparticles, Expert. Opin Drug Deliv.

[12] Tapiero H, Mathé G, Couvreur P, Tew KD (2002). L-arginine. Biomed Pharmacother.

[13] Wu G, Jaeger LA, Bazer FW, Rhoads JM (2004). Arginine deficiency in preterm infants: biochemical mechanisms and nutritional implications. J Nutr Biochem.

[14] Skipper A. Dietitian's Handbook of enteral and parenteral nutrition: Jones & Bartlett Learning; 1998. [Google Scholar].

[15] Wu QX, Lin DQ, Yao SJ (2014). Design of chitosan and its water soluble derivatives-based drug carriers with polyelectrolyte complexes. Marine Drugs.

[16] Hamman JH (2010). Chitosan based polyelectrolyte complexes as potential carrier materials in drug delivery systems. Marine Drugs.

[17] Onishi H, Machida Y (1999). Biodegradation and distribution of water-soluble chitosan in mice. Biomaterials.

[18] Xia WS, Liu P, Liu J (2008). Advance in chitosan hydrolysis by non-specific celluloses. Bioresour Technol.

[19] Zhang H, Alsarra IA, Neau SH (2002). An *in vitro* evaluation of a chitosan-containing multi-particulate system for macromolecule delivery to the colon. Int J Pharm.

[20] Xu PS, Bajaj G, Shugg T, van Alstine WG, Yeo Y (2010). Zwitterionic chitosan derivatives for pH-sensitive stealth coating. Biomacromolecules.

[21] Bajaj G, van Alstine WG, Yeo Y (2012). Zwitterionic chitosan derivative, a new biocompatible pharmaceutical excipient, prevents endotoxin-mediated cytokine release. PLoS One.

[22] Čalija B, Cekić N, Savić S, Daniels R, Marković B, Milić J (2013). pH-sensitive micro-particles for oral drug delivery based on alginate/oligo-chitosan/Eudragit®L100–55 “sandwich” polyelectrolyte complex. Colloid Surf B.

[23] Luo Y, Wang Q (2014). Recent development of chitosan-based polyelectrolyte complexes with natural polysaccharides for drug delivery. Int J Biol Macromol.

[24] Karande P, Mitragotri S (2009). Enhancement of transdermal drug delivery *via* synergistic action of chemicals, Biochim. Biophys Acta.

[25] Manosroi J, Apriyani MG, Foe K, Manosroi A (2005). Enhancement of the release of azelaic acid through the synthetic membranes by inclusion complex formation with hydroxypropyl-beta-cyclodextrin. Int J Pharm.

[26] Wang S, Tan M, Zhong Z, Chen M, Wang Y (2011). Nanotechnologies for curcumin: An ancient puzzler meets modern solutions. J Nanomater.

[27] Martin EM, Valle D (2004). Cyclodextrins and their uses: a review. Process Biochem.

[28] Odile DC, Blanca MV, Didier B (2004). Controlled ring-opening polymerization of lactide and glycolide. Chem Rev.

[29] Yoo DK, Kim D, Lee DS (2006). Synthesis of Lactide from Oligomeric PLA: Effects of Temperature, Pressure, and Catalyst. Macromol Res.

[30] Pourasghar M, Koenneke A, Meiers P, Schneider M (2019). Development of a fast and precise method for simultaneous quantification of the PLGA monomers lactic and glycolic acid by HPLC. J Pharm Anal.

[31] Matthews CE, Van Holde KE, Ahern KG (1999). Biochemistry.

[32] Green MM, Blankenhorn G, Hart H (1975). Which starch fraction is water-soluble, amylose or amylopectin. J Chem Educ.

[33] Wichterle O, Lím D (1960). Hydrophilic gels for biological use. Nature.

[34] Sung YK, Jhon MS, Gregonis DE, Andrade JD (1981). Thermal and pulse nuclear magnetic resonance analysis of water in poly (2-hydroxyethyl methacrylate). J Appl Polym Sci.

[35] Katt ME, Placone AL, Wong AD, Xu ZS, Searson PC (2016). In Vitro Tumor Models: Advantages, Disadvantages, Variables, and Selecting the Right Platform. Front Bioeng Biotechnol.

[36] Friedrich JS, Claudia ER, Kunz-Schughart LA (2009). Spheroid-based drug screen: considerations and practical approach. Nat Protoc.

[37] Ferreira L, Vidal MM, Gil MH (2000). Evaluation of poly (2-hydroxyethyl methacrylate) gels as drug delivery systems at different pH values. Int J Pharm.

[38] Hsiue GH, Cheng CC (2001). Poly(2-hydroxyethyl methacrylate) film as a drug delivery system for pilocarpine. Biomaterials.

[39] Hanak BW, Hsieh CY, Donaldson W, Browd SR, Lau KS, Shain W (2018). Reduced cell attachment to poly (2-hydroxyethyl methacrylate)-coated ventricular catheters *in vitro*. J Biomed Mater Res B Appl Biomater.

[40] Heskins M, Guillet JE (1968). Solution properties of poly(N-isopropyl acrylamide). J Macromol Sci Part A Chem.

[41] Zheng L, Qiulin L, Duanguang Y, Yong G, Xujun L (2013). Well-defined poly(*N*-isopropylacrylamide) with a bifunctional end-group: synthesis, characterization, and thermoresponsive properties, Designed. Monomers Polymers.

[42] Schmaljohann D (2006). Thermo- and pH-responsive polymers in drug delivery. Adv Drug Deliv Rev.

[43] Ma Y, Hou S, Ji B, Yao Y, Feng X (2010). A novel temperature-responsive polymer as a gene vector. Macromol Biosci.

[44] Weber C, Richard H, Schubert US (2012). Temperature responsive bio-compatible polymers based on poly (ethylene oxide) and poly (2-oxazoline)s. Prog Polym Sci.

[45] Bae YH, Okano T, Kim SW (1990). Temperature dependence of swelling of crosslinked poly(N,N′-alkyl substituted acrylamides) in water. J Polym Sci Part B Polym Phys.

[46] Okano T, Yamada N, Sakai H, Sakurai Y (1993). A novel recovery system for cultured cells using plasma-treated polystyrene dishes grafted with poly(N-isopropyl acrylamide). J Biomed Mater Res.

[47] Yoo MK, Sung YK, Lee YM, Cho CS (2000). Effect of polyelectrolyte on the lower critical solution temperature of poly(N-isopropyl acrylamide) in the poly (NIPAAm-*co*-acrylic acid) hydrogel. Polymer.

[48] Zhang Q, Ko NR, Oh IK (2012). Recent advances in stimuli-responsive degradable block copolymer micelles: synthesis and controlled drug delivery applications. Chem Commun.

[49] Tanaka R, Ueoka I, Takaki Y, Kataoka K, Saito S (1983). High molecular weight linear polyethylenimine and poly(N-methylethylenimine). Macromolecules.

[50] Weyts KF, Goethals EJ (1988). New synthesis of linear polyethyleneimine. Polym Bull.

[51] Brissault B, Kichler A, Guis C, Leborgne C, Danos O, Cheradame H (2003). Synthesis of linear polyethylenimine derivatives for DNA transfection. Bioconjug Chem.

[52] Yang J, Zhang R, Pan H, Li Y, Fang Y, Zhang L, Kopeček J (2017). Backbone degradable N-(2-hydroxypropyl) methacrylamide copolymer conjugates with gemcitabine and paclitaxel: Impact of molecular weight on activity toward human ovarian carcinoma xenografts. Mol Pharm.

[53] Zhang R, Yang J, Zhou Y, Shami PJ, Kopeček J (2016). N-(2-hydroxypropyl) methacrylamide copolymer–drug conjugates for combination chemotherapy of acute myeloid leukemia. Macromol Biosci.

[54] Pan H, Yang J, Kopeckova P, Kopecek J (2011). Backbone degradable multiblock *N*-(2-hydroxypropyl) methacrylamide copolymer conjugates *via* reversible addition−fragmentation chain transfer polymerization and thiolene coupling reaction. Biomacromolecules.

[55] Zhang L, Zhang R, Yang J, Wang J, Kopecek J (2016). Indium-based and iodine-based labeling of HPMA copolymer-epirubicin conjugates: Impact of structure on the *in vivo* fate. J Control Release.

[56] GilliesJean ER, Fréchet MJ (2005). Dendrimers and dendritic polymers in drug delivery. Drug Discov Today.

[57] Menjoge AR, Kannan RM, Tomalia DA (2010). Dendrimer-based drug and imaging conjugates: design considerations for nano-medical applications. Drug Discov Today.

[58] Nam HY, Nam K, Lee M, Kim SW, Bull DA (2012). Dendrimer type bio-reducible polymer for efficient gene delivery. J Control Release.

[59] Törmälä P, Pohjonen T, Rokkanen P (1998). Bio-absorbable polymers: materials technology and surgical applications. Proc Inst Mech Eng.

[60] Pulapura S, Kohn J (1992). Trends in the development of bio-resorbable polymers for medical applications. J Biomater Appl.

[61] Lee TS, Bee ST. Polylactic Acid (second edition), A practical guide for the processing, manufacturing and applications of PLA, plastics design library: Elsevier; 2019. p. 53–95. Elsevier B.V. [Google Scholar].

[62] Heller J, Barr J, YNg S, Abdellauoi KS, Gurny R (2002). Poly (ortho esters): synthesis, characterization, properties and uses. Adv Drug Deliv Rev.

[63] Kumar N, Langer RS, Domb AJ (2002). Polyanhydrides: an overview. Adv Drug Deliv Rev.

[64] Kamaly N, Yameen B, Wu J, Farokhzad OC (2016). Degradable controlled-release polymers and polymeric nanoparticles: Mechanisms of controlling drug release. Chem Rev.

[65] Nicolas J, Mura S, Brambilla D, Mackiewicz N, Couvreur P (2013). Design, functionalization strategies and biomedical applications of targeted biodegradable/biocompatible polymer-based nanocarriers for drug delivery. Chem Soc Rev.

[66] Kwon OJ, Kang E, Choi JW, Kim SW, Yun CO (2013). Therapeutic targeting of chitosan-PEG-folate-complexed oncololytic adenovirus for active and systematic cancer gene therapy. J Control Release.

[67] Choi JW, Nam JP, Nam K, Lee YS, Yun CO, Kim SW (2015). Oncolitic adenovirus coated with multi-degradable bio-reducible core-cross-linked poly-(ethyleneimine) for cancer gene therapy. Biomacromolecules.

[68] Choi JW, Kim HA, Nam K, Na Y, Yun CO, Kim SW (2015). Hepatoma targeting peptide conjugated bioreducible polymer complexed with oncolytic adenovirus for cancer gene therapy. J Control Release.

[69] Pack DW, Hoffman A, Pun S, Stayton PS (2005). Design and development of polymers for gene delivery. Nat Rev Drug Discov.

[70] Ray L, Polymeric nanoparticle-based drug/gene delivery for lung cancer, *in* nanotechnology-based targeted drug delivery systems for lung cancer, 2019; Chap. 4: 77–93 [Google Scholar].

[71] Pandey VN, Tiwari N, Pandey VS, Rao A, Das I (2019). Targeted drug delivery and gene therapy through natural biodegradable nanostructures in pharmaceuticals, in nanoarchitectonics in biomedicine.

[72] Gu Z. Bioinspired and biomimetic polymer systems for drug and gene delivery: Chemical Industry Press and Wiley-VCH Verlag GmbH & Co.; Wiley. KGaA; 2015. ISBN: 9783527334209 |Online ISBN: 9783527672752. [Google Scholar].

[73] Bagalkot V, Farokhzad OC, Langer R, Jon S (2006). An aptamer–doxorubicin physical conjugate as a novel targeted drug-delivery platform. Angew Chem Int Ed.

[74] Speck O, Speck D, Horn R, Gantner J, Sedlbauer KP (2017). Biomimetic bio-inspired biomorph sustainable. An attempt to classify and clarify biology-derived technical developments. Bioinspir Biomim.

[75] Vincent JF (2009). Biomimetics: a review. Proc Inst Mech Eng H.

[76] Sabua C, Rejob C, Kottab S, Pramoda K (2018). Bioinspired and biomimetic systems for advanced drug and gene delivery. J Control Release.

[77] Safari J, Zarnegar Z (2014). Advanced drug delivery systems: Nanotechnology of health design A review. J Saudi Chem Soc.

[78] Li Y, Thambi T, Lee DS (2018). Co-delivery of drugs and genes using polymeric nanoparticles for synergistic cancer therapeutic effects. Adv Healthcare Mater.

[79] Yoo JW, Irvine DJ, Discher DE, Mitragotri S (2011). Bio-inspired, bioengineered and biomimetic drug delivery carriers. Nature Rev Drug Disc.

[80] Yang L, Li J, Kopeček J (2019). Biorecognition: A key to drug-free macromolecular therapeutics. Biomaterials.

[81] Li J, Yang S, Soodvilai J, Wang P, Opanasopit J, Kopeček J (2019). Drug-free albumin triggered sensitization of cancer cells to anticancer drugs. J Control Release.

[82] Patra JK, Das G, Fraceto LF, Campos EVR, Rodriguez-Torres MP, Acosta-Torres LS, Diaz-Torres LA, Grillo R, Swamy MK, Sharma S, Habtemariam S, Shin HS (2018). Nano based drug delivery systems: recent developments and future prospects. J Nanobiotechnol.

[83] Li J, Yang J, Wang J, Kopeček J (2018). Drug-free macromolecular therapeutics exhibit amplified apoptosis in G2/M phase arrested cells. J Drug Target.

[84] Verma IM, Somia N (1997). Gene therapy-promises, problems and prospects. Nature.

[85] Yin H, Kanasty RL, Eltoukhy AA, Vegas AJ, Dorkin JR, Anderson DG (2014). Non-viral vectors for gene-based therapy. Nat Rev Genet.

[86] Beck-Broichsitter M, Merkel OM, Kissel T (2012). Controlled pulmonary drug and gene delivery using polymeric nano-carriers. J Control Release.

[87] Guo X, Huang L (2012). Recent advances in non-viral vectors for gene delivery. Acc Chem Res.

[88] Nam K, Jung S, Nam JP, Kim SW (2015). Poly (ethylenimine) conjugated bio-reducible dendrimer for efficient gene delivery. J Control Release.

[89] Sung YK, Kim SW (2019). Recent advances in the development of gene delivery systems. Biomater Res.

[90] Zhong Z, Feijen J, Lok MC, Hennink WE, Christensen LV, Yockman JW, Kim YH, Kim SW (2005). Low molecular weight linear polyethylenimine-*b*-poly (ethylene glycol)-*b*-polyethylenimine triblock copolymers: synthesis, characterization, and in vitro gene transfer properties. Biomacromolecules.

[91] Park MR, Han KO, Han IK, Cho MH, Nah JW, Choi YJ, Cho CS (2005). Degradable polyethylenimine-*alt*-poly (ethylene glycol) copolymers as novel gene carriers. J Control Release.

[92] Wen Y, Pan S, Luo X, Zhang X, Zhang W, Feng M (2009). A biodegradable low molecular weight polyethylenimine derivative as low toxicity and efficient gene vector. Bioconjug Chem.

[93] Wen Y, Pan S, Luo X, Zhang W, Shen Y, Feng M (2010). PEG- and PDMAEG-graft-modified branched PEI as novel gene vector: synthesis, characterization and gene transfection. J Biomater Sci Polym Ed.

[94] Kim SW, Nam JP, Kim S, Sung YK (2018). Recent development of bio-reducible polymers for efficient gene delivery system. J Cancer Treatment Diagn.

[95] Nam JP, Park JK, Son DH, Kim TH, Park SJ, Park SC, Choi C, Jang MK, Nah JW (2014). Evaluation of polyethylene glycol conjugated novel polymeric antitumor drug for cancer therapy. Colloids Surf B: Biointerfaces.

[96] Ou M, Wang XL, Xu R, Chang CW, Bull DA, Kim SW (2008). Novel Biodegradable Poly (disulfide amine) s for Gene Delivery with High Efficiency and Low Cytotoxicity. Bioconjug Chem.

[97] Nam JP, Kim S, Kim SW (2018). Design of PEI-conjugated bio-reducible polymer for efficient gene delivery. Int J Pharm.

[98] Lee YS, Kim SW (2014). Bioreducible polymers for therapeutic gene delivery. J Control Release.

[99] Rai R, Alwani S, Badea I (2019). Polymeric nanoparticles in gene therapy: New avenues of design and optimization for delivery applications. Polymers (Basel).

[100] Kim T, Kim SW (2011). Bioreducible polymers for gene delivery. React Funct Polym.

[101] Smith AE (1995). Virial vectors in gene therapy, viral vectors in gene therapy. Annu Rev Microbiol.

[102] Sung YK, Kim SW (2018). The practical application of gene vectors in cancer therapy. Integrat Cancer Sci Therap.

[103] Ibraheem D, Elaissari A, Fessi H (2014). Gene therapy and DNA delivery systems. Int J Pharm.

[104] Lundstrom K (2003). Latest development in viral vectors for gene therapy. Trends Biotechnol.

[105] Robbins PD, Ghivizzani SC (1998). Viral vectors for gene therapy. Pharmacol Therap.

[106] Nathaly S, Pere D, Anna C, Victor R, Salvador B (2014). Oligopeptide-terminated poly(β-amino ester) s for highly efficient gene delivery and intracellular localization. Acta Biomater.

[107] Usman WM, Pham TC, Kwok YY, Vu LT, Ma V, Peng B, Chan YS, Wei L, Chin SM, Azad A, He AB-L, Leung AYH, Efficient RNA (2018). drug delivery using red blood cell extracellular vesicles. Nat Commun.

[108] Wang F, Zhang W, Shen Y, Huang Q, Zhou D, Guo S, Efficient RNA (2015). delivery by integrin-targeted glutathione responsive polyethyleneimine capped gold nanorods. Acta Biomater.

[109] Whitehead KA, Langer R, Anderson DG (2008). Knocking down barriers: advances in siRNA delivery. Nat Rev Drug Discov.

[110] Arthanari Y, Pluen A, Rajendran R, Aojula H, Demonacos C (2010). Delivery of therapeutic shRNA and siRNA by tat fusion peptide targeting BCR-ABL fusion gene in chronic myeloid leukemia cells. J Control Release.

[111] Davis ME, Zuckerman JE, Choi CH, Seligson D, Tolcher A, Alabi CA (2010). Evidence of RNAi in humans from systemically administered siRNA *via* targeted nanoparticles. Nature.

[112] Merritt WM, Bar-Eli M, Sood AK (2010). The dicey role of dicer: implications for RNAi therapy. Cancer Res.

[113] Mathiyalagan P, Sahoo S (2017). Exosomes-based gene therapy for micro-RNA delivery methods. Mol Biol.

[114] Zhang D, Lee H, Zhu Z, Minhas JK, Jin Y (2017). Enrichment of selective miRNAs in exosomes and delivery of exosomal miRNAs *in vitro* and *in vivo*. Am J Phys Lung Cell Mol Phys.

